# Office Visitors

**Published:** 1906-08

**Authors:** 


### OFFICE VISITORS.

He was a very well dressed young man of twenty-five years probably, who greeted us in our reception room with a good deal of warmth. "Why, howdy do, doctor, possibly you do not recognize me, for the first time I met you was at my father's house in I——, in the mountains, years ago, when you were summering there—that is if you are the same Dr. T——." We assured him we were the same individual and remembered the kind hospitality of his father very vividly. "Ah, yes, sir, he was a good man, too good, doctor, for his own good, for he died only a few years after your visit and left his family quite poorly off; yes, sir, quite poorly off, and we've had a hard time battling with this world. Yes, we left the old place years ago, after his death, and have struggled along in the city of A——. It has indeed been a struggle with us. But, dear doctor, I am glad to say, though one of the youngest children, I've been fortunate and grateful that it has been my lot to help the family along. Yes, sir, I have a good position as a traveling salesman for a good firm at a good salary. Yes, doctor, a very good salary, and I am able to do very well by them. I have some little trouble, doctor, with my gums, which I get treated along the road as I travel, and would you please look at them." We did so, and in consideration of the former acquaintance with his family and their kindness in the years gone by, treated a slight gingivites, free of charge. He was profuse in his thanks and appeared so genuine, notwithstanding a certain air of cant and hypocrisy about him, that we invited him into our laboratory that we might talk to him as we work. We went over all the old times at T—— and he gave us much information as to the old inhabitants and acquaintances both among the living and the dead. The next day he appeared again and walked right into our work room, without announcement, and then we began to be suspicious. The climax was reached when he practically informed us that he was gone broke on the town and had failed to get a remittance due within the last twelve hours, but if I would identify him at the city bank he could have a check cashed. We saw through the game and cooling turning in our seat and looking him in the eye informed him that we were up to his proposition and appreciated him as a degenerate member of a very worthy family and that now his absence would be enjoyed more than his presence. He slunk out like a cur with his tail between his legs and we found out that he had tried his confidence game on several others.

